# Effect of biosurfactants on *Pseudomonas aeruginosa* and *Staphylococcus aureus* biofilms in a BioFlux channel

**DOI:** 10.1007/s00253-016-7310-5

**Published:** 2016-01-29

**Authors:** M. A. Diaz De Rienzo, P. S. Stevenson, R. Marchant, I. M. Banat

**Affiliations:** School of Chemical Engineering and Analytical Science, University of Manchester, Manchester, M13 9PL UK; Unilever Research and Development Laboratory, Port Sunlight, Wirral CH62 4ZD UK; School of Biomedical Sciences, Faculty of Life and Health Sciences, University of Ulster, Coleraine, BT52 1SA Northern Ireland, UK

**Keywords:** Biosurfactants, Biofilms, Rhamnolipids, BioFlux

## Abstract

Recent studies have indicated that biosurfactants play a role both in maintaining channels between multicellular structures in biofilms and in dispersal of cells from biofilms. A combination of caprylic acid (0.01 % *v*/*v*) together with rhamnolipids (0.04 % *v*/*v*) was applied to biofilms of *Pseudomonas aeruginosa* ATCC 15442, *Staphylococcus aureus* ATCC 9144 and a mixed culture under BioFlux flowthrough conditions and caused disruption of the biofilms. The biofilms were also treated with a combination of rhamnolipids (0.04 % *v*/*v*) and sophorolipids (0.01 %). Control treatments with PBS 1× had no apparent effect on biofilm disruption. The Gram-positive bacterium (*S. aureus* ATCC 9144) was more sensitive than *P. aeruginosa* ATCC 15442 in terms of disruption and viability as shown by Live/Dead staining. Disruption of biofilms of *P. aeruginosa* ATCC 15442 was minimal. Oxygen consumption by biofilms, after different treatments with biosurfactants, confirms that sophorolipid on its own is unable to kill/inhibit cells of *P. aeruginosa* ATCC 15442, and even when used in combination with rhamnolipids, under static conditions, no decrease in the cell viability was observed. Cells in biofilms exposed to mono-rhamnolipids (0.04 % *v*/*v*) showed behaviour typical of exposure to bacteriostatic compounds, but when exposed to di-rhamnolipids (0.04 % *v*/*v*), they displayed a pattern characteristic of bactericidal compounds.

## Introduction

Deposition of microorganisms on solid surfaces and subsequent biofilm formation are phenomena that happen naturally but are also part of the microorganisms’ strategy to protect themselves from external toxic factors. Intercellular signalling, often referred to as quorum sensing, has been shown to be involved in biofilm development by several bacteria, including *Pseudomonas aeruginosa*, *Burkholderia cepacia*, *Streptococcus mutans*, and others (Yarwood et al. 2004). *P. aeruginosa* biofilms have been extensively studied due to their relative ease of formation under various conditions in vitro, their medical importance and the genetic tractability of the organism involved (Irie et al. 2005). In addition, infections caused by community-acquired (CA) methicillin-resistant *S. aureus* (MRSA) have been reported worldwide. Such infections pose a serious problem frequently in hospitalized patients and healthcare personnel. Although many anti-staphylococcal antibiotics have been developed, treatment options for MRSA infections are limited as a result of the emergence of antibiotic resistance (Samadi et al. 2012).

Biosurfactants of microbial origin have been reported to have antiadhesive and biofilm disruption abilities (Banat et al. 2014). In recent years, rhamnolipids derived from *P. aeruginosa* have emerged as an important group of biosurfactants with several applications (Marchant and Banat 2012a, b); they have also been produced on a commercial scale (Dusane et al. 2010). During the later stages of *P. aeruginosa* biofilm development, the production of the biosurfactant rhamnolipids was shown to be important in modulating microcolony architecture by maintaining channels to allow fluids to flow through the biofilm. However, the mechanisms by which the bacteria maintain these channels once they form have not been investigated. Davey et al. (2003) indicated that *P. aeruginosa* not only regulates development of its distinctive biofilm architecture but also, once channels form, this organism utilizes rhamnolipid surfactants to actively maintain the void spaces surrounding macrocolonies; in other words, they propose that rhamnolipids are not required for the formation of macrocolonies and channels but participate in the maintenance of channels once they are formed.

Antimicrobial properties are not restricted just to rhamnolipids, biosurfactants produced by *Lactobacilli*; other strains have also been shown to reduce adhesion of pathogenic micro-organisms to glass, silicone rubber and surgical implants (Fracchia et al. 2010; Quinn et al. 2013). Therefore, prior adsorption of biosurfactants can be used as a preventive strategy to delay the onset of pathogenic biofilm growth on catheters and other medical materials, reducing the use of synthetic drugs and chemicals. Another possible strategy to reduce the global problem of antimicrobial resistance is the combination of two or more compounds together, which has been used in recent years (Samadi et al. 2012). In this continuing search for new combinations of natural products as antimicrobial agents, we found that biosurfactants produced by *P. aeruginosa* in combination with caprylic acid have potential activity against pre-formed biofilms of *S. aureus* and *P. aeruginosa.* We also investigated the effects mono-rhamnolipids and di-rhamnolipids have on *P. aeruginosa* cells in biofilms.

## Materials and methods

### Microorganisms and culture conditions

*P. aeruginosa* ATCC 15442 and *S. aureus* ATCC 9144 were acquired from the American Type Culture Collection (ATCC). The strains were maintained in nutrient broth plus 20 % glycerol at −20 °C. Bacterial growth from a nutrient agar slant incubated for 24 h at 30 °C was used to obtain a bacterial suspension with an optical density at 570 nm adjusted to give 10^8^ CFU/mL.

### Rhamnolipids characteristics

The rhamnolipid containing 10 % (*w*/*v*) mono-rhamnolipid (C_26_H_48_O_9_, MW 504, critical micelle concentration (CMC) 20 mg/L M at neutral pH) and 10 % (*w*/*v*) di-rhamnolipid (C_32_H_58_O_13_, MW 650, CMC 1.5 × 10^−4^ 30 mg/L at neutral pH) was obtained from Jeneil Biosurfactant Co. (Saukville, WI, USA). Mono-rhamnolipids and di-rhamnolipids were obtained as described by Rudden et al. (2015) and kept refrigerated at 4 °C until further use.

### Sophorolipid characteristics

The sophorolipid SL18 containing 50 % hydrophile (sophorose) plus 50 % lipophile (fatty acid), with density 1.1 ± 0.1, a water activity 35 ± 5 %, surface tension 38 mN/m, and CMC 40 mg/L, was obtained from ECOVER (Newbury-Berkshire, UK).

### Biofilm growth on the BioFlux flowthrough device

To analyze biofilm formation under flow conditions, the BioFlux 200 system (Fluxion Biosciences Inc., South San Francisco, CA) was used which allows automated image acquisition within specialized multiwell plates. To grow biofilms, the microfluidic channels (depth, 75 μm; width, 350 μm) were primed with TSB (50 %) at 10.0 dyn/cm^2^. Channels were seeded with 10^7^ CFU from an overnight culture of *P. aeruginosa* ATCC 15442, *S. aureus* ATCC 9144 and a mixed culture of both. The plate was then incubated at 30 °C for 48 h to allow cells to adhere. After biofilms had formed, planktonic cells were removed, and PBS 1× (as control) and different treatments were added to the input wells at a flow rate of 279 μL/h for 30 min. The results were recorded with a microscope Evon (×10) (17 % light).

### Growth of “static” biofilms on OxoPlates®

*P. aeruginosa* ATCC 15442 was grown overnight as previously described and diluted 100-fold with TSB 50 % following which a 100-μL sample of each diluted culture was dispensed (eight replicates) to fill a 96-well OxoPlate OP96C®. After 48 h, each well was rinsed twice with PBS 1×S, and the active different treatments were added to each well during 30 min at 30 °C. OxoPlate OP96C (PreSens, Regensburg, Germany) contains oxygen-sensitive particles PSLi-Pt-1 (Opto-Sense, Wörth, Germany), which consist of small polystyrene particles. The sensor has a thickness of about 10 μm and is fixed at the bottom of each well of a 96-flat bottom-well plate (Greiner, Frickenhausen, Germany). The oxygen concentration in each well was followed for 21 h at 20-min intervals. Fluorescence of each well was measured in dual kinetic mode (BMG Labtech GmbH, Germany). Filter pair 1 (544/650 nm) detects fluorescence of the indicator dye. The second filter pair (544/590 nm) measures fluorescence of the reference dye.

All experiments were repeated on separate days. Oxygen concentration as percentage air saturation was calculated for each well by using the following equation :

$$ {pO}_2=100\cdotp \left(\frac{k_0}{I_R}-1\right)/\left(\frac{k_0}{k_{100}}-1\right) $$

## Results

### Biofilm disruption of *P. aeruginosa* ATCC 15442, *S. aureus* ATCC 9144 and a mixed culture using rhamnolipids and caprylic acid

**Fig. 1:**
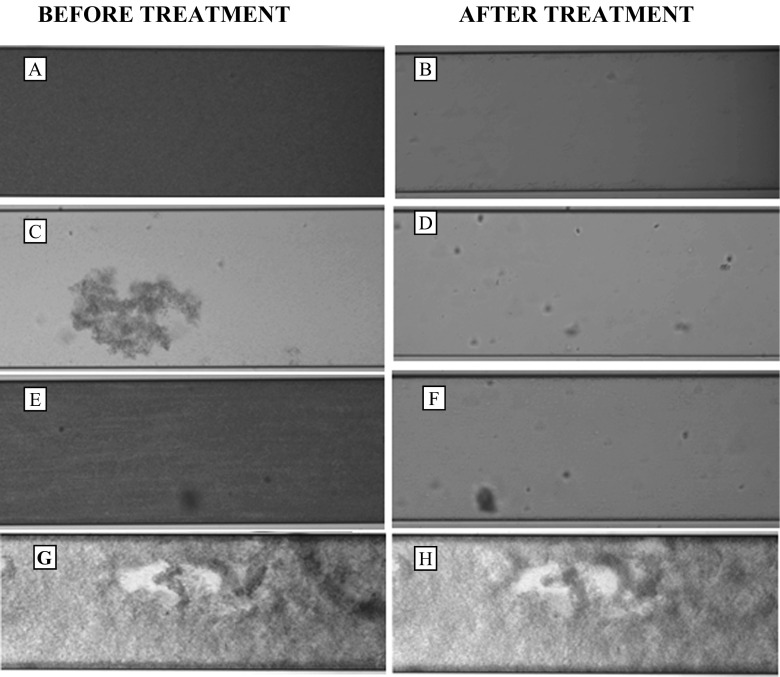
Biofilm formation and disruption in a BioFlux channel. The images are phase-contrast images and show fully formed biofilms after 48 h of incubation at 30 °C, and the images were recorded with a microscope Evon (10×) (17 % light). **a** *P. aeruginosa* ATCC 15442 biofilm before treatment. **b** *P. aeruginosa* ATCC 15442 after treatment with rhamnolipid (0.04 %) and caprylic acid (0.01 %). **c** *S. aureus* ATCC 9144 before treatment. **d** *S. aureus* ATCC 9144 after treatment with rhamnolipid (0.04 %) and caprylic acid (0.01 %). **e** Mixed culture (*P. aeruginosa* ATCC 15442/*S. aureus* ATCC 9144). **f** Mixed culture after treatment with rhamnolipid (0.04 %) and caprylic acid (0.01 %). **g** *P. aeruginosa* ATCC 15442 before treatment. **h** *P. aeruginosa* ATCC 15442 after treatment with PBS 1×

The effect of rhamnolipids together with caprylic acid on pre-formed biofilms by *P. aeruginosa* ATCC 15442, *S. aureus* ATCC 9144 and a mixed culture was determined under BioFlux flowthrough conditions. The disruption produced by the combination of caprylic acid together with rhamnolipids was confirmed. All isolates developed biofilms over 48 h. However, there was considerable variability in terms of spread around of the microfluidic channel. *P. aeruginosa* biofilms and the mixed culture were well formed (Fig 1a, e, g) under flow conditions; however, the biofilms formed by *S. aureus* ATCC 9144 (Fig. 1c) were not as thick, but good enough to be considered a multicellular community that represented a fundamentally different physiological state compared to free-living planktonic bacteria.

After 48 h, all the plates were rinsed with PBS 1× (a water-based salt solution in which the osmolarity and ion concentrations of the solution) as a control, and the treatment combinations of rhamnolipids (0.04 % *v*/*v*) and caprylic acid (0.01 % *v*/v) were applied for 30 min, after which period more than the 90 % disruption of the biofilms had occurred. It is interesting to note that *P. aeruginosa* ATCC 15442 and *S. aureus* ATCC 9144 respond differently to the combination between rhamnolipids and caprylic acid, compared to individual application (Dusane et al. 2010, Dusane et al. 2012), suggesting a possible interaction between rhamnolipids and caprylic acid.

### Effect of rhamnolipids and sophorolipids on biofilm disruption of *P. aeruginosa* ATCC 15442, *S. aureus* ATCC 9144 and a mixed culture

Previous studies have shown that rhamnolipids can bring about disruption of pre-formed biofilms of *P. aeruginosa* and *S. aureus* and indicate that sophorolipids have potential to be used individually for efficient removal and killing of detrimental biofilms formed by *Bacillus subtilis* (Díaz De Rienzo et al. 2015a). To investigate different combinations between natural compounds for the treatment of infections caused by multidrug-resistant bacteria, we used a combination between rhamnolipids and sophorolipids for the disruption/killing of preformed biofilms of *P. aeruginosa* ATCC 15442 and *S. aureus* ATCC 9144.

**Fig. 2:**
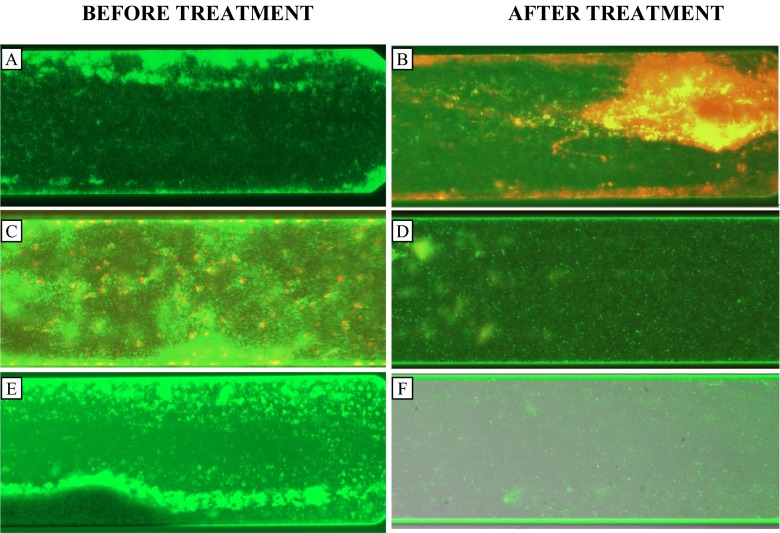
Effect of rhamnolipids and sophorolipids on Biofilm disruption. Biofilms were grown for 48 h at 30 °C and then stained with Live/Dead BacLight and the images were recorded with a microscope Evon (×10) (17 % light). **a** *P. aeruginosa* ATCC 15442 biofilm before treatment. **b** *P. aeruginosa* ATCC 15442 after treatment with rhamnolipid (0.04 %) and sophorolipid (0.01 %). **c** *S. aureus* ATCC 9144 before treatment. **d** *S. aureus* ATCC 9144 after treatment with rhamnolipid (0.04 %) and sophorolipid (0.01 %). **e** Mixed culture (*P. aeruginosa* ATCC 15442/*S. aureus* ATCC 9144). **f** Mixed culture after treatment with rhamnolipid (0.04 %) and sophorolipid (0.01 %)

The treatment of the all the biofilms with PBS 1× had no apparent effects on the biofilm, and all the cells were well established and viable (Fig. 2a, c, e). The Gram-positive bacterium *S. aureus* ATCC 9144 was more sensitive to the combination of rhamnolipids and sophorolipids (Fig 2d) than *P. aeruginosa* ATCC 15442 (Fig. 2b), not just in terms of the disruption but in terms of viability as shown by LIVE/DEAD staining. Interestingly, biofilm disruption of *S. aureus* ATCC 9144 and the mixed culture was apparently not caused by the reported bactericidal activity of rhamnolipids or sophorolipids, because the majority of the remaining attached cells (Fig. 2d, f) were viable (green stained). On the other hand, the disruption of biofilms of *P. aeruginosa* ATCC 15442 (Fig. 2B) was minimal; after 30 min of treatment, just a few cells were detached as shown by the images before and after treatment. However, the remaining cells were stained red, which indicated that this combination at the concentrations evaluated is not effective in removing the cells but is effective in killing them. This could mean that the combination between rhamnolipids (0.04 %) and sophorolipids (0.01 %) may be used as a specific strategy to kill cells within biofilms of *P. aeruginosa* ATCC 15442 within a period on 30 min.

### Effect of different biosurfactants on oxygen consumption by biofilms of *P. aeruginosa* ATCC 15442

Biofilms of *P. aeruginosa* ATCC 15442 were formed on an OxoPlate® after 48 h at 30 °C; once the biofilms were rinsed with PBS 1× twice, they were incubated for half an hour under different treatments (Table 1).

**Table 1:** Biosurfactant treatments applied prior to the measurement of the oxygen consumption of biofilms of *Pseudomonas aeruginosa* ATCC 15442

Treatment	Concentration (*v*/*v*)^a^
PBS 1×	–
Sophorolipids	0.01 %
Rhamnolipids/sophorolipids	0.04 %**/**0.01 %
Mono-rhamnolipids	0.04 %
Di-rhamnolipids	0.04 %

^a^All concentrations used are above the critical micelle concentrations for these biosurfactants

**Fig. 3:**
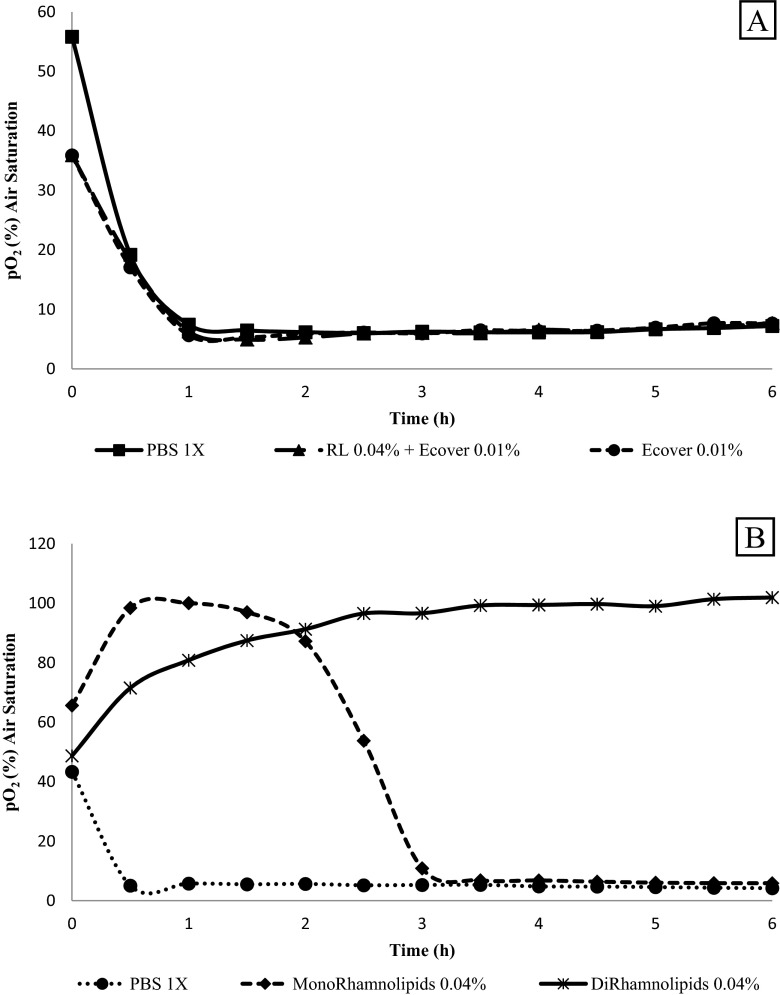
Oxygen consumption of *P. aeruginosa* ATCC 15442 biofilms after 30-min treatment with **a** combinations of biosurfactants and **b** mono- and di-rhamnolipids. Shown in a plot of the relative concentration of dissolved oxygen in percentage of saturation concentration versus time after addition of the different substances. Treatment concentrations are indicated

A minimum number of cells were required to consume a threshold amount of oxygen before they were detected in the system. All the results shown in Fig. 3a are above this threshold due to a high inoculum density; as a consequence, consumption of oxygen was detected immediately and the growth medium was essentially free of oxygen after 1 h, for all treatments used. These results confirm that sophorolipids on their own are unable to kill or inhibit oxygen uptake by cells of *P. aeruginosa* ATCC 15442, and even in combination with rhamnolipids, under static conditions, no decrease in the cell metabolism was observed.

As described above, oxygen consumption was quantified for different treatments including mono- and di-rhamnolipids separately at 0.04 % *v*/*v*. In Fig. 3b, the curves also give insight into the kinetics of bacterial growth inhibition. The cells with mono-rhamnolipid treatment showed a delay of oxygen consumption during the first 2 h of active growth; however, the oxygen level in each well declined gradually to 0 % and stayed at this level, behaviour typical of bacteriostatic compounds. On the other hand, the cells treated with di-rhamnolipids displayed a strikingly dissimilar pattern, because of the initial effect after the treatment when the oxygen concentration increased. This increase is attributable to the enhanced diffusion of atmospheric oxygen into the wells after cell death, which is an indication that we are dealing with a bactericidal compound.

## Discussion

Understanding the complex way that bacteria colonize and build specialized structures like biofilms and formulating new strategies to deal with their formation or facilitate their disruption through removal or killing are current issues in medical and industrial microbiology. One of the possible solutions for this global problem is the appropriate use of antimicrobial combinations (Menichetti 2005). The results of biofilm disruption of *P. aeruginosa* ATCC 15442, *S. aureus* ATCC 9144 and a mixed culture using rhamnolipids and caprylic acid showed that this strategy is effective under flow cell conditions, not just for cell removal but also for killing most of the cell population (data not shown). This is true for either *P. aeruginosa* ATCC 15442, *S. aureus* ATCC 9144 or a mixed culture of both, despite the fact that most bacterial biofilms display resistance against antimicrobials such as antibiotics and various host immune responses (Jesaitis et al. 2003).

Rhamnolipids are secreted by *P. aeruginosa* and are involved in biofilm formation through the promotion of motility, the inhibition of attachment and the degradation of the matrix maintaining channels throughout the biofilm for movement of water and oxygen (Banat et al. 2014); however, when the biofilm is already formed, the external addition of rhamnolipids could be involved in the removal of extracellular polymeric substances (EPS) and destruction of microcolonies altering the biofilm environment as a result of their surface active properties, in addition to a generalized activity of altering charge-charge properties (Davey et al. 2003), which may decrease the chances for the bacteria to acquire resistance due to spontaneous mutations. In this report, the combination between rhamnolipids and caprylic acid induced disruption of mature biofilms of *P. aeruginosa* ATCC 15442, *S. aureus* ATCC 9144 and a mixed culture, despite the fact that most of the *P. aeruginosa* ATCC 15442 cells were alive, showing that the primary effect of this combination does not appear to be bactericidal.

The use of rhamnolipids with other biosurfactants may have a strong ability to disrupt biofilm structures, in the same way that was already shown for combination with antibiotic treatments (Samadi et al. 2012). In this study, we show that the combination between rhamnolipids and sophorolipids could be useful to remove cells of *S. aureus* ATCC 9144 and a mixed culture between them, previously attached to a polymeric surface. Nevertheless, when we consider the effect on biofilms of *P. aeruginosa* ATCC 15442, the effect of this combination is different, due to the fact that most of the cells were dead after the treatment. The combination between rhamnolipids and sophorolipids at the concentration evaluated in this work might be used as a specific strategy to kill or eliminate biofilms of *P. aeruginosa* ATCC 15442 under flow conditions, while the bioactivity and possible synergistic interactions and effect between these biosurfactant need to be further investigated.

The mechanism for bioactivity of biosurfactants is suggested to be associated with their intercalation into target cell membranes. Samadi et al. (2012) demonstrated that rhamnolipids (as a mixture of mono and di forms) and mono-rhamnolipids and di-rhamnolipids separately were more effective against Gram-positive than Gram-negative bacteria such as *P. aeruginosa* when they are growing in the planktonic form, due to the fact that the lipopolysaccharide (LPS) present in the outer membrane increases the negative charge of the cell membrane and its lipid portion is impermeable to the charged rhamnolipids. Several methods are in use to quantify bacterial growth in the presence or absence of antibacterial compounds, to study planktonic and biofilm behaviour of diverse populations of cells. Here we used a fluorescence assay system that quantifies the oxygen concentration in the growth medium called OxoPlates®. This study showed how mono- and di-rhamnolipids have an antimicrobial effect on biofilms by *P. aeruginosa* ATCC 15442, measured as a decrease of oxygen concentration over time (Fig. 3a, b) on OxoPlates®; however, the effect of rhamnolipids, a mixture of mono and di forms, was not evaluated on oxoplates but they were confirmed as potential disruptors in previous experiments (Díaz De Rienzo et al. 2015b).

The cells treated with mono-rhamnolipids and di-rhamnolipids showed different mechanisms of action. Mono-rhamnolipids act as bacteriostatic compounds, and the effect of di-rhamnolipids is bactericidal as indicated by the response that we observed in Fig. 3b, related to oxygen consumption. Rhamnolipids are biologically produced compounds and assumed to be biocompatible and safe for human use; recent investigations by our group at Ulster University showed that acidic sophorolipids induced a dose-dependent decrease in cell viability of colorectal cancer cell lines without affecting the viability of the colonic epithelial and lung cell lines (Callaghan et al. 2015). However, their effects in vivo are yet to be established. Research is therefore undergoing to test the different effects of selected rhamnolipid congeners on both normal and cancer cell lines.

Further studies should focus on the action of different natural rhamnolipids either alone or in combination with other compounds like antibiotics or enzymes which play an important role in the stability of the EPS during biofilm formation (Meers et al. 2008). This may open a new approach to combat the establishment or possible disruption of biofilm formation by different bacterial species through disruption of adhesion or growth of coated medical and industrial instruments, for example. It would however be important to take into account that combinations with other compounds described above may behave differently for particular species. In this study, we have confirmed the biofilm disruption of *P. aeruginosa* ATCC 15442, *S. aureus* ATCC 9144 and a mixed culture using rhamnolipids and caprylic acid, as well as the greater sensitivity of *S. aureus* ATCC 9144 over *P. aeruginosa* in terms of disruption and viability as shown by Live/Dead staining. In addition, a combination of rhamnolipids and sophorolipids might be used as a specific strategy to eliminate biofilm of *P. aeruginosa* ATCC 15442 under flow conditions, as a result of a bacteriostatic or bactericidal mechanism of action.
